# Salivary Enzyme-Responsive Switching Nanoparticles Overcoming Diffusion and Absorption Barriers for Transmucosal Delivery

**DOI:** 10.1021/acsami.6c05144

**Published:** 2026-06-03

**Authors:** Se Kye Park, Lam Tan Hao, Hee Jung Park, Dong Yun Lee, Hyeonyeol Jeon, Hyo Jeong Kim

**Affiliations:** † Research Center for Bio-Based Chemistry, 65680 Korea Research Institute of Chemical Technology (KRICT), Ulsan 44429, Republic of Korea; ‡ Department of Polymer Science and Engineering, 34986 Kyungpook National University, Daegu 41566, Republic of Korea; § Technical Support Center for Chemical Industry, Korea Research Institute of Chemical Technology (KRICT), Ulsan 44429, Republic of Korea; ∥ Advanced Materials & Chemical Engineering, Korea National University of Science and Technology (UST), Daejeon 34113, Republic of Korea

**Keywords:** buccal drug delivery, salivary enzyme-responsive, mucoadhesion, mucus penetration, guanidinium chitosan oligosaccharide

## Abstract

Transmucosal drug delivery is an attractive noninvasive strategy, yet its efficiency is fundamentally limited by the trade-off between mucus penetration and mucoadhesion. Here, we present salivary enzyme-responsive penetration-to-adhesion switching nanoparticles (SEPAS NPs) designed to overcome this limitation in the context of buccal drug delivery. SEPAS NPs feature a dual-regime architecture consisting of an *N*-guanidinium chitosan oligosaccharide (G-COS) inner layer that provides mucoadhesion and a degradable starch-based outer layer that transiently shields cationic interactions with mucins during transport. Under mucus-mimicking conditions, SEPAS NPs exhibit minimal mucin binding during penetration, followed by enzyme-mediated degradation of the starch shell that exposes the underlying G-COS layer and enhances retention at a mucin-coated interface by approximately 6-fold. Furthermore, this switching design does not compromise drug release, maintaining diffusion-dominated sustained release of dexamethasone (DEX). In addition, SEPAS NPs demonstrate excellent cytocompatibility (>90% cell viability) and maintain the anti-inflammatory activity of released DEX. Collectively, this work presents an enzyme-responsive nanoparticle strategy that resolves the penetration–adhesion trade-off while maintaining favorable release kinetics and biocompatibility, providing a broadly adaptable platform for transmucosal drug delivery across diverse mucus environments.

## Graphical abstract

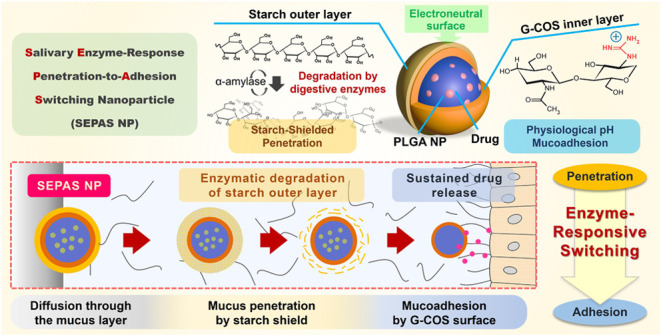

## 1 Introduction

Buccal drug delivery is a compelling noninvasive alternative to parenteral administration because it can enable rapid systemic absorption through the highly vascularized oral mucosa while avoiding gastrointestinal degradation and hepatic first-pass metabolism.^1–3^ Owing to its easy accessibility and relatively thin epithelial barrier, the buccal route has attracted increasing attention for both local therapy and systemic delivery, particularly for pediatric, geriatric, and noncooperative patients for whom swallowing can be challenging.^4–7^

Nanoparticle-based formulations offer additional advantages for buccal administration by protecting labile drugs, enabling controlled release, and allowing surface engineering to modulate interactions with the mucosa.^8–10^ Poly­(lactic-*co*-glycolic acid) (PLGA) is widely used as a biocompatible and biodegradable nanoparticle (NP) matrix that enables sustained drug release, surface modification, and protection of encapsulated drugs from degradation.^11,12^ However, in transmucosal delivery, PLGA NPs require additional surface engineering to overcome the physiological barriers imposed by the mucosal environment. The buccal mucus layer, which is formed by the cross-linking and entanglement of mucins, impedes NP diffusion through electrostatic interactions and steric hindrance within the mucus mesh.^13–15^ Concurrently, continuous salivary secretion, involuntary swallowing, and frequent oral movements rapidly remove exogenous materials, severely limiting the residence time of formulations.^16,17^ Consequently, an efficient buccal nanocarrier must possess (i) mucus penetrating capability to reach the epithelium and (ii) mucoadhesive capability to remain localized near the epithelial interface long enough to support drug absorption. These two requirements are often difficult to satisfy simultaneously.

Mucus penetrating particles (MPPs) have been extensively investigated to enhance NP diffusion through the mucus layer. Representative approaches include dense surface shielding with hydrophilic and nonionic polymers (e.g., poly­(ethylene glycol, poloxamers)),^18–20^ enzymes (e.g., papain, trypsin),^21^ and cell penetrating peptides (e.g., penetratin).^22,23^ However, these mucus-inert surfaces typically lack the ability to anchor near the epithelial interface and are therefore prone to rapid removal under salivary flow. Cationic mucoadhesive materials such as chitosan can improve retention by forming electrostatic and hydrogen-bonding interactions with negatively charged mucins.^24–26^ However, conventional chitosan exhibits pH-dependent protonation of its amino groups (p*K*_a_ of amino group ≈ 6–7), which leads to reduced charge density under near-physiological buccal conditions (pH ≈ 6.3), resulting in reduced adhesion and drug delivery performance.^27^ Guanidinium-functionalized chitosan derivatives have been explored to strengthen mucin binding because guanidinium groups remain protonated at near-neutral pH and form more stable interactions with mucosal components.^28–30^ While stronger mucoadhesion can improve initial retention, highly charged surfaces also tend to form adhesive interactions with mucins that immobilize nanoparticles in the superficial mucus layer, limiting epithelial access and ultimately reducing delivery efficiency. Collectively, these observations highlight an intrinsic penetration-retention trade-off that continues to constrain buccal nanoparticle delivery.^31–33^

Here, we report salivary enzyme-responsive penetration-to-adhesion switching nanoparticles (SEPAS NPs) that sequentially implement mucus transport and epithelial anchoring through an α-amylase-triggered interfacial transition. SEPAS NPs comprise a guanidinium-rich inner layer of *N*-guanidinium chitosan oligosaccharide (G-COS) to enable strong mucoadhesion at the epithelial interface, and a degradable starch outer layer that initially masks the cationic surface to facilitate mucus penetration (Figure 1a). As schematically illustrated in Figure 1b, SEPAS NPs are incorporated into an adhesive buccal patch to support mucus penetration followed by localized mucoadhesion near the epithelial target site. The starch outer layer is progressively degraded by salivary enzymes such as α-amylase, exposing the cationic G-COS layer and inducing a transition from mucus penetration to mucoadhesion. In contrast, conventional NP-based buccal delivery systems face a penetration-retention trade-off (Figure 1c): unmodified PLGA NPs show poor epithelial retention, whereas permanently cationic G-COS-coated nanoparticles are susceptible to mucin-mediated trapping within the mucus layer. Using complementary transport and retention assessments, together with drug delivery simulations and *in vitro* evaluation, we demonstrate that SEPAS NPs enhance epithelial access while maintaining prolonged residence, leading to improved therapeutic performance. Unlike conventional designs that optimize either penetration or adhesion in isolation, SEPAS provides an enzyme-programmable route to combine both functions within a single nanosystem. These findings suggest that the SEPAS strategy can be extended beyond buccal delivery to a broad range of mucus-associated applications, where mucus barriers fundamentally limit drug transport and therapeutic efficacy.

**Figure 1:**
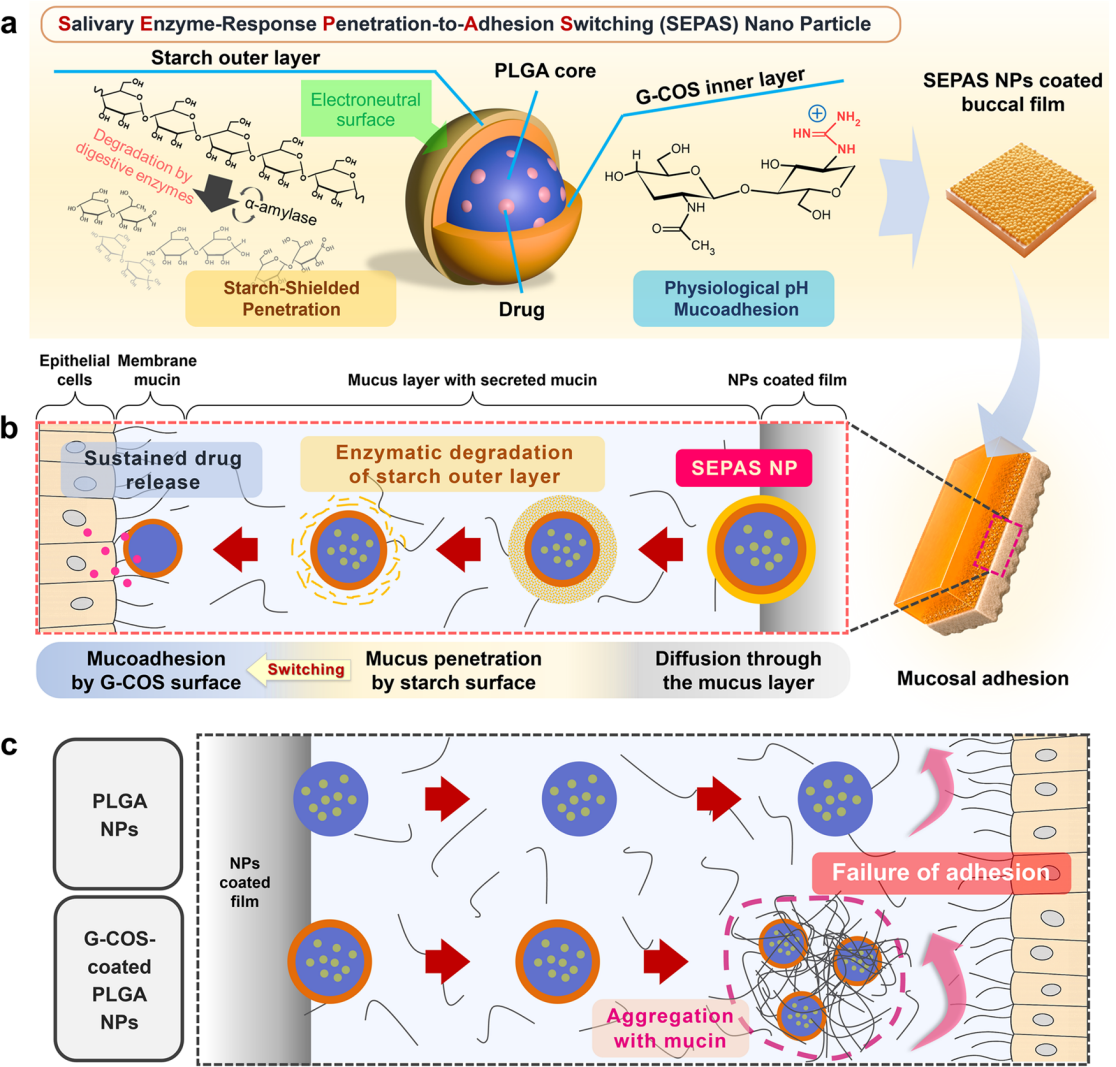
Schematic illustration of salivary enzyme-responsive penetration-to-adhesion switching nanoparticles (SEPAS NPs) for buccal drug delivery. (a) Structural design of SEPAS NPs consisting of a G-COS inner layer and a starch outer layer. The G-COS coating maintains a positive charge at biological pH to enable strong adhesion to epithelial cells, while the starch outer layer masks the cationic G-COS surface and is degradable by salivary enzymes. (b) Salivary enzyme triggered penetration-to-adhesion switching of SEPAS NPs in the buccal mucosa. (c) Limitations of conventional PLGA NPs and permanently cationic G-COS-coated PLGA NPs in the buccal mucosal environment, including insufficient epithelial retention and mucin-mediated trapping, respectively.

## 2 Experimental Section

### 2.1 Materials

Chitosan oligosaccharide (COS) was purchased from Tokyo Chemical Industry (Japan). Scandium­(III) triflate, cyanamide, acetic acid, PLGA, Poly­(vinyl alcohol) (PVA, *M*_w_ 89–98 kDa), dexamethasone (DEX, ≥98%), zinc acetate (99.99%), ammonium persulfate (APS, ≥98%), sodium dodecyl sulfate (SDS, ≥98.5%), 2-mercaptoethanol (99%), acetone (≥99.8%), methanol (≥99.8%), Nile red (for fluorescence microscopy), mucin (from porcine stomach, Type III), α-Amylase (from human saliva, 300–1,500 units mg^–1^ protein), phosphate buffered saline (PBS, pH 7.2–7.6), *N*-(3-(Dimethylamino)­propyl)-*N*′-ethylcarbodiimide hydrochloride, sucrose (≥99.5%), Tween 20, bromophenol blue sodium salt, heat-shock bovine serum albumin (BSA, pH 7, ≥98%), lipopolysaccharide (LPS, *Escherichia coli* O111:B4), and monoclonal anti-β-actin antibody (mouse) were obtained from Sigma-Aldrich (USA). Starch (for nanoparticle coating) was obtained from SAMCHUN (Korea). (Heptadecafluoro-1,1,2,2-Tetrahydrodecyl)­Triethoxysilane were purchased from Gelest (USA). Sulfo-NHS (*N*-hydroxysulfosuccinimide) and Alexa Flour 488 were purchased from Thermo Fisher Scientific (USA). Low-density polyethylene (LDPE) strips (USP reference standard) were used as the negative control. Radioimmunoprecipitation assay (RIPA) buffer, cOmplete^TM^ Mini protease inhibitor cocktail tablets, and SuperSignal West Pico PLUS chemiluminescent substrate were purchased from Thermo Fisher Scientific (USA). Horseradish peroxidase-conjugated AffiniPure goat antimouse IgG (H + L) was obtained from Jackson ImmunoResearch (USA). Mouse anti-COX-2 (H-3) antibody was purchased from Santa Cruz Biotechnology (USA). Acrylamide/Bis solution (29:1, 30%) and Tris–HCl buffers (1.5 M, pH 6.8 and 8.8) were purchased from Biosesang (South Korea). Griess reagent nitrite assay kit, Tris–glycine SDS running buffer (10×), Tris–glycine transfer buffer (10×), and Tris-buffered saline (TBS, 10×) were obtained from Cell Signaling Technology (USA).

### 2.2 Synthesis of *N*-Guanidinium Chitosan

G-COS was synthesized by guanidinylation of COS using cyanamide in the presence of scandium­(III) triflate under acidic conditions, following a reported method.^34^ COS (2 wt %) was dispersed in 2 wt % aqueous acetic acid, followed by addition of cyanamide (3.782 mmol) and scandium­(III) triflate (0.189 mmol). The reaction mixture was refluxed at 100 °C for 48 h. The product was precipitated with acetone, filtered, washed with acetone, and vacuum-dried at 80 °C for 24 h (Figure S1). Prior to characterization and use, polysaccharide powders were redispersed in water and lyophilized (≤−40 °C; freeze-dryer, Ilshin Biobase, South Korea).

### 2.3 Preparation of G-COS/Starch Dual-Coated PLGA Nanoparticles

DEX-loaded, SEPAS NPs were prepared by a modified nanoprecipitation method (Figure S2).^35^ PLGA and DEX were dissolved in acetone as the organic phase. G-COS was dissolved in an aqueous PVA solution, where PVA served as a temporary stabilizer to provide steric stabilization during nanoparticle formation. The organic phase was added dropwise into the aqueous phase under stirring (300 rpm) at a feed rate of 0.5 mL min^–1^ to form G-COS-coated PLGA NPs. After solvent evaporation, nanoparticles were collected by centrifugation (11,000 rpm, 30 min) and washed with water to remove PVA and unbound species. The outer starch layer was introduced by incubating the G-COS coated NP pellet in a dilute starch solution (0.1 w/v%), followed by an additional washing step. Nanoparticles were cryoprotected with sucrose and lyophilized.

### 2.4 Characterization

Particle morphology was examined by SEM (Sigma 300 VP, Carl Zeiss, Germany). Hydrodynamic diameter (Z-average), polydispersity index (PDI), and ζ-potential were measured by DLS using a Zetasizer Advance (Malvern, U.K.). ^1^H NMR spectra were recorded using 500 MHz liquid-state NMR spectrometer (AvanceNeo500, Bruker, USA). Samples were prepared at concentrations of 10–15 mg mL^–1^ in DMSO-*d*_6_/D_2_O (9:1, v/v). FTIR spectra of NPs were acquired by an infrared spectrometer (Nicolet iS50, Thermo Scientific, USA) at 400–4000 cm^–1^ using the attenuated-total-reflection mode. Drug loading was quantified by extracting DEX from purified nanoparticles in a DMSO/phosphate buffer mixture (9:1) and analyzing by UV–Vis spectroscopy (V-770, Jasco, USA) using a calibration curve. The encapsulation efficiency (EE) and drug loading capacity (DLC) were calculated using standard mass-balance equations

1 $$\mathrm{EE}\left(\%\right)=\frac{\mathrm{weight}\;\mathrm{of}\;\mathrm{drug}\;\mathrm{in}\;\mathrm{particles}}{\mathrm{weight}\;\mathrm{of}\;\mathrm{particles}}\times 100$$

2 $$\mathrm{DLC}\left(\%\right)=\frac{\mathrm{weight}\;\mathrm{of}\;\mathrm{loaded}\;\mathrm{drug}}{\mathrm{total}\;\mathrm{weight}\;\mathrm{of}\;\mathrm{the}\;\mathrm{drug}\;\mathrm{loaded}}\times 100$$

### 2.5 Mucin Binding Efficiency

Nanoparticle–mucin interactions were evaluated using a mucin binding assay. Mucin (0.5 mg) was dispersed in 1 mL of deionized water, and nanoparticles (NPs) were dispersed at 0.3 mg/mL in 2 mL of deionized water. The two solutions were mixed at a 1:2 (v/v) ratio and incubated at 37 °C for 60 min under gentle shaking. After incubation, the samples were centrifuged at 8000 rpm for 30 min. The concentration of noninteracted mucin in the supernatant was determined by UV–vis spectroscopy at 261 nm, using a mucin solution without NPs as the control. The mucin binding efficiency was calculated as

3 $$m\mathrm{ucin binding efficiency}\left(\%\right)=\frac{A_{control}-A_{sample}}{A_{control}}\times 100$$

Where *A*_control_ represents the absorbance of mucin solution without NPs and *A*_sample_ denotes the absorbance of supernatant after incubation with NPs. The resulting NP–mucin aggregates (pellet) were redispersed in PBS, and their ζ-potentials were measured to assess surface charge changes induced by mucin binding.

### 2.6 Evaluation of Nanoparticle Interactions with the Mucin Layer

#### 2.6.1 Qualitative Analysis of Nanoparticle Interaction with Mucin Layer

Fluorescent dye conjugation to mucin was carried out using EDC/sulfo-NHS chemistry.^36^ The fluorescent dye (Alexa flour 488) was first diluted to a concentration of 1.0 mg mL^–1^ in 0.01 M MES buffer (pH 5.0) to a final volume of 1 mL. Subsequently, EDC (5 × 10^–3^ M) and sulfo-NHS (0.01 M) were added to the solution, which was then incubated for 3 h at room temperature under light-protected conditions to activate the carboxyl groups for subsequent conjugation to mucin. Separately, mucin (40 mg) was dissolved in 19 mL of phosphate-buffered saline (PBS, 0.01 M, pH 7.0). The activated Alexa Flour 488 solution was then mixed thoroughly with the mucin solution and allowed to react for 3 h at room temperature. Following conjugation, the reaction mixture was dialyzed against deionized water using a dialysis membrane with a molecular weight cutoff (MWCO) of 10 kDa for 2–3 days to remove unreacted dye and reagents. The purified fluorescently labeled mucin was subsequently lyophilized and stored for further use.

Glass slides were cut into 10 mm × 20 mm pieces and cleaned with toluene. The cleaned slides were treated with air plasma at 100 W for 30 s to activate the surface. Immediately after plasma treatment, the slides were exposed overnight to the vapor of 1,1,2,2-tetrahydrooctyl-1-trichlorosilane to render the surface hydrophobic via silanization to facilitate stable mucin adsorption. Mucin was dissolved in PBS (pH 7.4) at a concentration of 5 mg mL^–1^. The silanized glass slides were immersed in the mucin solution and incubated at 37 °C for 2 h with gentle agitation to allow mucin adsorption. The mucin-coated slides were then rinsed gently with PBS to remove loosely bound mucin prior to further experiments.

A mucin-coated slide glass prepared using Alexa Flour 488-conjugated mucin was placed in a 35 mm Petri dish. Solution A was prepared by mixing mucin (0.6 mg mL^–1^) with α-amylase (60–300 IU mL^–1^) in PBS. Solution B was prepared by dispersing Nile Red-encapsulated nanoparticles at a concentration of 4 mg mL^–1^ in PBS.

Solutions A and B were mixed at a 1:1 volume ratio to obtain a total volume of 5 mL, vortexed for 10 s, and then added to the Petri dish containing the mucin-coated slide. The Petri dish was incubated in a shaking incubator at 37 °C for 24 h. After incubation, the glass slide was removed, thoroughly washed with PBS to eliminate nonadherent nanoparticles, and observed using a fluorescence microscope.

#### 2.6.2 Quantitative Analysis of Nanoparticle Adhesion

For quantitative evaluation, mucin was coated onto the wells of a well plate following the same coating procedure described for the qualitative analysis. Artificial saliva was added to each well to simulate the mucosal environment (Table S1).^37^ Subsequently, 40 μL of Nile Red-encapsulated nanoparticle dispersion (prepared in PBS with or without α-amylase) was added to each well.

The well plate was incubated in a shaking incubator at 37 °C for 24 h. After incubation, each well was gently washed with PBS to remove nonadherent nanoparticles. The fluorescence intensities of Alexa Fluor 488 (mucin) and Nile Red (nanoparticles) were then measured using a microplate reader to quantify nanoparticle adhesion to the mucin layer.

### 2.7 In Vitro Drug Release Study

Five mg of DEX-loaded NPs was dispersed in 1 mL of PBS (pH 7.4) containing α-amylase (60–300 IU mL^–1^). The NP suspension was transferred into a dialysis bag with a molecular weight cutoff of 12–14 kDa. The dialysis bag was immersed in 70 mL of PBS (pH 7.4) as the receptor medium and incubated at 37 °C under gentle shaking. At predetermined time points of 5, 15, 30, 60, 120, 240, and 480 min, and 12, 24, and 48 h, 1 mL of the release medium was withdrawn and replaced with an equal volume of fresh PBS to maintain sink conditions. The amount of released DEX was quantified using High Performance Liquid Chromatography (ACQUITY ARC; Waters, USA) with a methanol/water mobile phase (65:35, v/v) and flow rate of 1.0 mL min^–1^. All experiments were performed in triplicate (*n* = 5).

### 2.8 Cell Handling

NIH-3T3 fibroblasts and RAW264.7 macrophages were obtained from the American Type Culture Collection (ATCC, https://www.atcc.org/). Cells were grown in DMEM supplemented with FBS (10 v/v%) and antibiotics (100 U mL^–1^ penicillin and 100 μg mL^–1^ streptomycin). Cultures were incubated at 37 °C in a humidified atmosphere containing 5% CO_2_ and subcultured before reaching 80% confluence. Cells with a passage number below 10 were used for all experiments.

### 2.9 In Vitro Cytotoxicity

The cytotoxicity of NP components was evaluated following ISO-10993–5 standard using CCK-8. Freeze-dried powder of NP components (0.2 g) was incubated in growth medium (1 mL) at 37 °C for 24 h with shaking (100 rpm) to obtain the test extract. Growth medium, LDPE strip extract (0.2 g mL^–1^) and zinc acetate (500 μM) were used as the blank, negative and positive controls, respectively.

Cells (10 μL, 10^5^ cells mL^–1^) were seeded into a 96-well plate containing a growth medium (90 μL) and incubated for 24 h. The medium was then replaced with the test extracts (100 μL) and incubated for another 24 h. After rinsing with DPBS (three times), cells were incubated in DMEM (100 μL, without phenol red) containing 1 mg mL^–1^ WST-8 reagent at 37 °C for 3 h. Viable cells reduced the WST-8 salt to orange formazan, which was quantified by measuring absorbance at 450 nm (optical density OD 450) using a microplate reader (Hidex Sense, Finland).^38^ Cell viability relative to the blank was calculated using eq 4.

$$\mathrm{viability}\left(\%\right)=\frac{{\mathrm{OD}}_{\mathrm{sample}}}{{\mathrm{OD}}_{\mathrm{blank}}}\times 100$$ 4

where OD_sample_ and OD_blank_ are the measured (OD 450–OD 650) values of the sample and blank, respectively. The 650 nm wavelength was subtracted to correct for interference from precipitated proteins and cellular debris.

### 2.10 Anti-Inflammatory Activity of Released DEX

#### 2.10.1 Pro/Antiinflammation Stimulation

RAW264.7 cells (10 μL, 10^5^ cells mL^–1^) were seeded into 96-well plates containing a growth medium (90 μL). After 24 h incubation, the culture medium was replaced with fresh culture medium alone (100 μL, blank control), or medium containing either LPS alone (proinflammatory control), LPS + DEX (antiinflammatory control), or LPS + NP dialysate. Cells were incubated for an additional 24 h. LPS was applied at 100 ng mL^–1^, and DEX was applied at 1 μM (DEX1) or 10 μM (DEX10). NP dialysates were collected as described in the drug release experiment except that the receptor phase volume of DPBS was reduced 10-fold. The dialysates were obtained after 12 h (NP12), 24 h (NP24), or 48 h (NP48) and diluted ten times in DMEM for cell treatment. Cells were incubated for an additional 24 h prior to downstream assaying.

#### 2.10.2 Griess Assay

To quantify NO production, each culture supernatant (40 μL) was transferred to a new 96-well plate and sequentially mixed with Griess reagent A (30 μL, 1 w/v% 4-aminosulfanilamide) and Griess reagent B [30 μL, 0.1% w/v *N*1-(naphthalen-1-yl)­ethane-1,2-diamine]. NO produced by RAW264.7 cells is rapidly converted to nitrite (NO_2_^–^), which reacts with Griess reagents to form an azo compound, whose absorbance at 550 nm was measured using a microplate reader. Nitrite concentrations were calculated using a sodium nitrite (NaNO_2_) standard calibration curve in the 1.5625–100 μM range.

#### 2.10.3 Gel Electrophoresis and Western Blot

RAW264.7 cells (10^6^ cells per well) were cultured in a six-well plate and subject to pro/antiinflammatory stimulation as described above. After aspirating the culture medium, cells were washed with ice-cold DPBS (three times) and lysed with RIPA buffer (200 μL per well) at 4 °C for 1 h. Cell lysates were centrifuged at 15,000 rpm for 15 min at 4 °C to collect the protein supernatants. Total protein concentrations were quantified using the Bradford assay, with bovine serum albumin (BSA, 125–1500 μg mL^–1^) as a standard.

Proteins were denatured in Laemmli buffer at 95 °C for 5 min, then separated via SDS–polyacrylamide gel electrophoresis using a 10 w/v% gel. Electrophoresis was performed at 120 V for 1.5 h following an initial run at 90 V for 15 min, using a Mini–PROTEAN Tetra Vertical Cell (Bio–Rad Laboratories, USA). Separated proteins were transferred to a methanol-activated poly­(vinylidene fluoride) membrane (LC2002, Thermo Fisher Scientific, USA) using a Mini Trans–Blot Cell (Bio–Rad Laboratories, USA) at 100 V for 1 h in an ice/water cooling bath.

After transfer, the membrane was blocked with 3 wt % BSA in Tris-buffered saline (pH 7.6) containing Tween 20 (TBST), followed by overnight incubation at 4 °C with mouse-derived primary antibodies including monoclonal anti-β-actin and anti-Cox-2. The membrane was then rinsed three times (10 min each) with TBST, incubated with a horseradish peroxidase-conjugated AffiniPure goat antimouse IgG (H + L) secondary antibody for 1 h at room temperature, and rinsed six more times (10 min each) with TBST. All antibodies were diluted in TBST containing 3 wt % BSA to the following final concentrations: primary antibodies (β-actin: 0.2 μg mL^–1^; Cox-2:0.5 μg mL^–1^), and secondary antibodies (β-actin: 0.02 μg mL^–1^, Cox-2:0.05 μg mL^–1^).

Immunoreactive blots were visualized by incubating the membrane with SuperSignal West Pico PLUS chemiluminescent substrate containing an enhancer for 5 min. Chemiluminescent signals were captured using a Gel Doc XR System (Bio–Rad Laboratories, USA). Protein expression levels were quantified by measuring blot intensities using Image Lab (Bio–Rad Laboratories, USA).

### 2.11 Statistical Analysis

All quantitative experiments were performed in triplicate (*n* = 3) using independently prepared samples, unless otherwise stated. Representative images, including SEM, AFM, and fluorescence microscopy images, were selected from at least three independent experiments. For comparative analyses, statistical significance was determined using a two-tailed Student’s *t* test. Statistical significance levels are indicated by asterisks: *, **, and ***, representing *p*-values of <0.05, < 0.01, and <0.001, respectively. Data are presented as mean ± standard deviation.

## 3 Results and Discussion

### 3.1 Preparation and Characterization of SEPAS NPs for Buccal Delivery System

SEPAS NPs were prepared to establish a dual-regime interfacial structure and systemically characterized for their physicochemical and drug-loading properties. G-COS was synthesized as the inner adhesive layer, and successful guanidinium functionalization of COS was confirmed prior to nanoparticle assembly (Figures S3–S5). The morphology and colloidal characteristics of PLGA, COS-coated PLGA (C–CP), G-COS-coated PLGA (G-CP), and SEPAS NPs were examined by SEM, AFM, and DLS (Figure 2a–c). SEM images revealed that pristine PLGA nanoparticles exhibited uniform spherical morphologies, whereas polysaccharide coating led to slightly increased particle size and less homogeneous surface features. AFM topographic images further indicated increased surface roughness after chitosan-based coating, which may be attributed to dehydration-induced collapse of the hydrophilic coating layer and heterogeneous surface coverage in the dried state (Figure 2b). In aqueous media (PBS), coated nanoparticles exhibited a time-dependent increase in hydrodynamic diameter accompanied by broadening of the size distribution (Figure 2c), suggesting progressive hydration of the polysaccharide layers and possible weak interparticle association over time. FTIR spectra of coated nanoparticles were consistent with successful introduction of chitosan-based layers (Figures 2d and S5). C–CP exhibited characteristic peaks of COS at 1652 cm^–1^, attributed to N–H bending, and at 1080 cm^–1^, corresponding to C–O stretching. In contrast, G-CP and SEPAS additionally exhibited a C=N stretching band at 1510 cm^–1^, which is consistent with the introduction of guanidinium groups, as reported in previous studies.^39–43^ Together, these results demonstrate that stepwise coating alters the surface morphology, chemistry, and colloidal behavior of PLGA nanoparticles.

**Figure 2:**
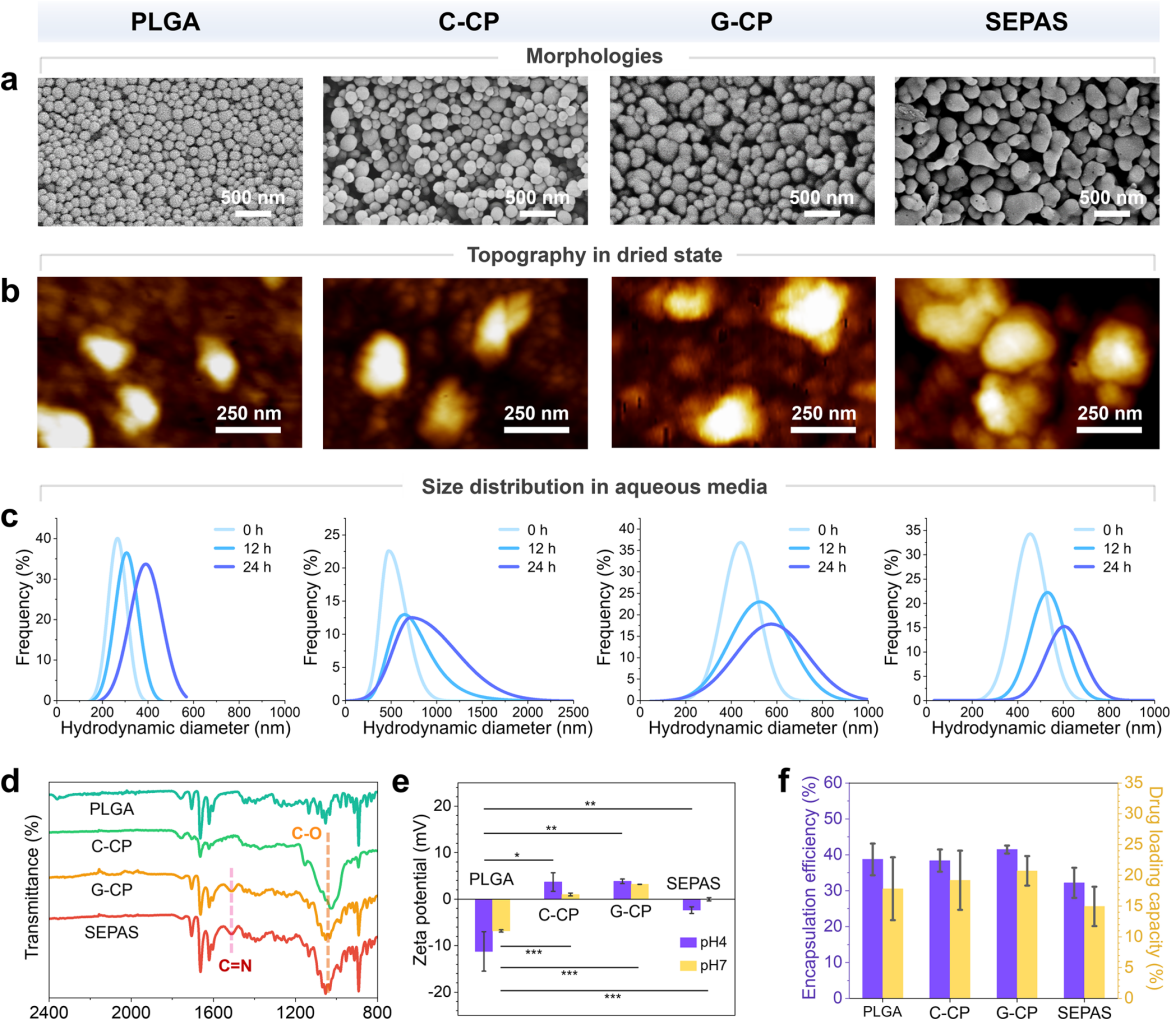
Physicochemical characterization of NPs. (a) SEM images of PLGA, C–CP, G-CP, and SEPAS NPs. (b) AFM images showing the surface morphology of the corresponding NPs. (c) DLS profiles of NPs after incubation and hydration in PBS for 0, 12, and 24 h; each profile represents the averaged frequency distribution (*n* = 3). (d) FT-IR spectra of PLGA, C–CP, G-CP, and SEPAS NPs. (e) ζ-potential of PLGA, C–CP, G-CP, and SEPAS NPs measured at pH 4 and pH 7. (f) Encapsulation efficiency (EE) and drug loading capacity (DLC) of the PLGA, C–CP, G-CP, and SEPAS NPs. Data in panels (e, f) are expressed as mean ± standard deviation (*n* = 3). Statistical significance between the indicated groups was analyzed using two-tailed Student’s *t* tests.

The ζ-potential of the individual shell components was first measured to confirm their intrinsic charge characteristics (Figure S6). COS showed a weak positive ζ-potential, G-COS exhibited a stronger positive ζ-potential, and starch showed a weak negative ζ-potential. These component-level charge properties were reflected in the stepwise surface modification of PLGA NPs (Figure 2e). Pristine PLGA NPs were negatively charged at both pH 4 and pH 7, consistent with ionization of terminal carboxyl groups on the NPs surface. C–CP became positively charged at pH 4 but approached near-neutral values at pH 7, reflecting the pH-dependent protonation of chitosan. In contrast, G-CP maintained a distinct positive potential under both pH values, confirming that guanidinium groups remain strongly cationic under near-physiological conditions. Notably, SEPAS showed near-neutral ζ-potentials at both pH 4 and 7, indicating effective charge masking of the underlying guanidinium-rich layer by the starch outer layer. This charge-masking behavior was supported by the concentration-dependent reduction in ζ-potential upon starch coating (Figure S7). Overall, these surface charge profiles define two distinct interfacial states within a single platform: an initially mucin-inert, transport-favorable shielded surface and a latent cationic layer that can mediate strong mucoadhesion upon unmasking, forming the physicochemical basis for penetration-to-adhesion switching.

To examine whether the stepwise shell assembly affected DEX retention within the PLGA core, DEX loading was evaluated across all formulations.^44^ As shown in Figure 2f, PLGA, C–CP, G-CP, and SEPAS NPs showed comparable EE (30–42%) and DLC (12–22%) values. Since DEX is codissolved with PLGA in the organic phase during nanoprecipitation and encapsulated within the polymer matrix prior to dual-layer coating, the observed variations likely reflect processing-related factors including particle recovery efficiency and mass balance changes, rather than direct modulation of core drug encapsulation by the coating materials. Importantly, SEPAS NPs retained substantial DEX loading after sequential G-COS and starch coating, confirming that the shell assembly process did not cause substantial drug leakage from the PLGA core and that the switchable surface architecture preserves drug reservoir integrity.

Finally, because buccal drug delivery is commonly achieved by coating drugs or nanocarriers onto thin film substrates, we evaluated the feasibility of coating SEPAS NPs onto gelatin films. Nile red–loaded NPs were deposited onto gelatin films for visualization (Figures S8–S11). The coating mass increased linearly with the applied nanoparticle amount (Figure S8), and SEM confirmed dense and uniform nanoparticle coverage on the film surface (Figure S9). Consistently, higher nanoparticle loading resulted in more intense macroscopic coloration (Figure S10) and a lower water contact angle than that of bare gelatin films (Figure S11), indicating increased surface hydrophilicity. These results collectively demonstrate that SEPAS NPs can be coated onto gelatin films in a tunable manner, suggesting their practical feasibility for buccal delivery systems.

### 3.2 Overcoming the Transmucosal Barrier via Penetration-to-Adhesion Switching

To evaluate the proposed SEPAS mechanisms, we first quantified nanoparticle–mucin affinity in buffered solutions at pH 4 and pH 7 (Figure 3a). Pristine PLGA nanoparticles exhibited negligible mucin binding, whereas both C–CP and G-CP showed markedly higher binding efficiencies, consistent with electrostatic interactions between cationic polymers and mucin.^44,45^ At pH 7, C–CP exhibited reduced binding relative to pH 4, reflecting the pH-dependent protonation of chitosan. In contrast, G-CP maintained high mucin binding at both pH values, supporting persistent mucin affinity mediated by guanidinium groups. Notably, SEPAS exhibited near-baseline mucin binding comparable to PLGA, indicating that the starch shield effectively masks the underlying guanidinium-rich layer and minimizes premature mucin-mediated aggregation and sequestration.

**Figure 3:**
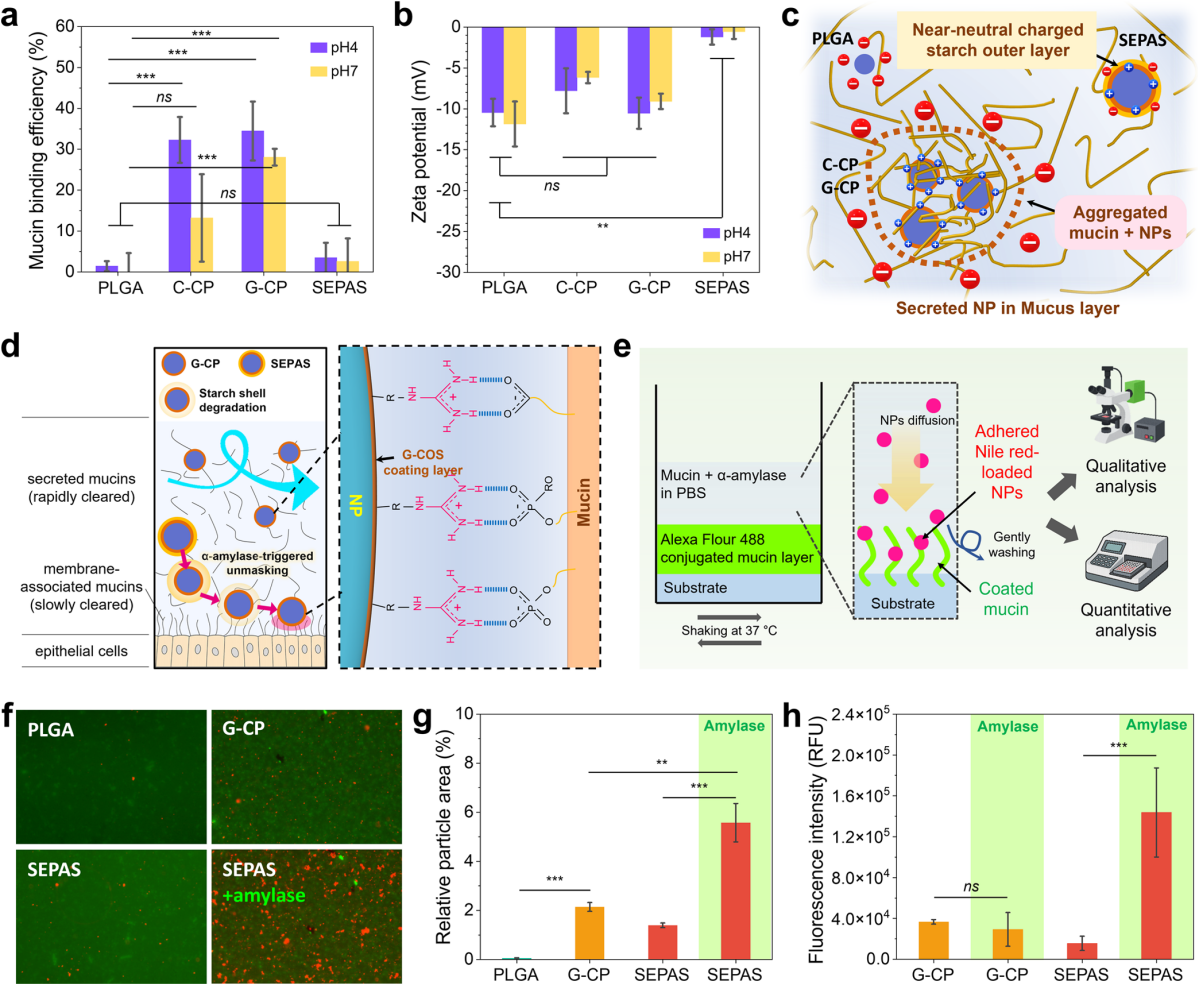
Transmucosal barrier overcoming by penetration-to-adhesion switching of SEPAS NPs. (a) Mucin binding efficiency of PLGA, C–CP, G-CP, and SEPAS. (b) ζ-potential of nanoparticle–mucin aggregates; PLGA, C–CP, G-CP, and SEPAS. (c) Schematic of mucin–nanoparticle interactions: C–CP and G-CP form aggregates with mucin, while SEPAS avoids mucin binding. (d) Schematic illustration of NP behavior within the buccal mucosal environment. (e) Experimental design for assessing penetration-to-adhesion switching of SEPAS NPs on mucin-coated substrates. (f) Fluorescence microscopy images of adhered PLGA, G-CP, and SEPAS NPs, showing SEPAS results in the presence and absence of α-amylase. (g) Quantification of adhered nanoparticles from fluorescence microscopy images, shown as the percentage of red fluorescent area. (h) Quantitative analysis of NP adhesion using a microplate reader to measure fluorescence intensity or Nile red-loaded NPs. Data are expressed as mean ± standard deviation (*n* = 3). Statistical significance between the indicated groups was analyzed using two-tailed Student’s *t* tests.

Consistently, ζ-potential measurements of nanoparticle–mucin mixtures suggested distinct interfacial states after mucin exposure (Figure 3b). Whereas C–CP and G-CP shifted from positive surface potentials (Figure 2e) to mucin-dominated negative values after incubation, consistent with mucin adsorption/aggregation dominating the outer interface (Figure 3c), PLGA remained negatively charged due to its intrinsic surface charge. In contrast, SEPAS remained near-neutral, supporting a charge-shielded, mucin-inert surface state.

Figure 3d schematically summarizes the hypothesized behavior of nanoparticles within the transmucosal barrier. G-CP NPs readily bind to secreted mucins, thereby restricting transport toward the epithelial cells and promoting clearance within the mucus layer. In contrast, SEPAS NPs, protected by a charge-shielding starch outer layer, exhibit minimal interaction with mucins, thereby facilitating access to the membrane-associated mucins. Upon salivary enzyme-mediated degradation of the starch outer layer, SEPAS NPs transitions into a mucoadhesive state by exposing the cationic G-COS layer, enabling programmable anchoring at the mucosal interface.

To test this switching mechanism, we examined whether SEPAS can transition from a mucus penetrating to a mucoadhesive state under salivary α-amylase conditions (Figure 3e). Alexa Flour 488 loaded nanoparticles were incubated in a soluble mucin environment, with or without α-amylase, above a mucin-coated substrate labeled with Alexa Fluor 488. Nanoparticle retention on the substrate was evaluated qualitatively and quantitatively.

Figure 3f shows representative fluorescence micrographs of retained NPs on the mucin-coated substrate. PLGA exhibited minimal retained deposition. Although G-CP possesses strong mucin affinity, it showed low net deposition on the underlying interface, likely because strong interactions with dispersed mucins promote sequestration into aggregates that are subsequently removed during washing. Importantly, SEPAS exhibited low deposition in the absence of amylase but a pronounced increase in retained deposition in the presence of amylase. Image-based quantification confirmed a multifold increase in the retained particle area for SEPAS upon amylase treatment (Figure 3g). This amylase-dependent enhancement indicates that SEPAS remains transport-favorable under mucin-rich conditions while selectively activating adhesion upon enzymatic unmasking.

Plate-reader quantification further corroborated this enzyme-triggered switching behavior (Figure 3h). G-CP showed no meaningful amylase-dependent enhancement, consistent with the absence of an enzyme-degradable shield. In contrast, SEPAS displayed a strong amylase-dependent increase in retained fluorescence intensity, demonstrating that starch degradation enables unmasking of the guanidinium-rich G-COS layer and converts SEPAS from a transport-favorable state to a strongly mucoadhesive state at the mucin-coated interface.

Together, these results support the SEPAS design, in which distinct NP transport and anchoring functions are sequentially integrated within a single system: (i) reduced mucin affinity to avoid premature immobilization during transport and (ii) salivary α-amylase-triggered unmasking to achieve robust mucoadhesion at the target interface, thereby addressing the penetration-retention trade-off that limits conventional buccal nanocarriers.

### 3.3 Drug Release Behavior and Kinetic Modeling of SEPAS

Figure 4a shows the *in vitro* release profiles of DEX from PLGA, C–CP, G-CP, and SEPAS NPs in a simulated buccal mucus environment (pH 6.8) containing α-amylase and mucin. The release behavior was monitored for 48 h to capture the tendency of the nanoparticle-mediated release profile, including the initial burst, the subsequent sustained-release phase and plateau under mucus-mimicking conditions. All formulations exhibited a biphasic release pattern characterized by an initial burst within the first 2–4 h followed by a slower, sustained release phase. PLGA and C–CP displayed comparable release kinetics, indicating that the COS coating does not markedly hinder drug release under these mucus-mimicking conditions.

**Figure 4:**
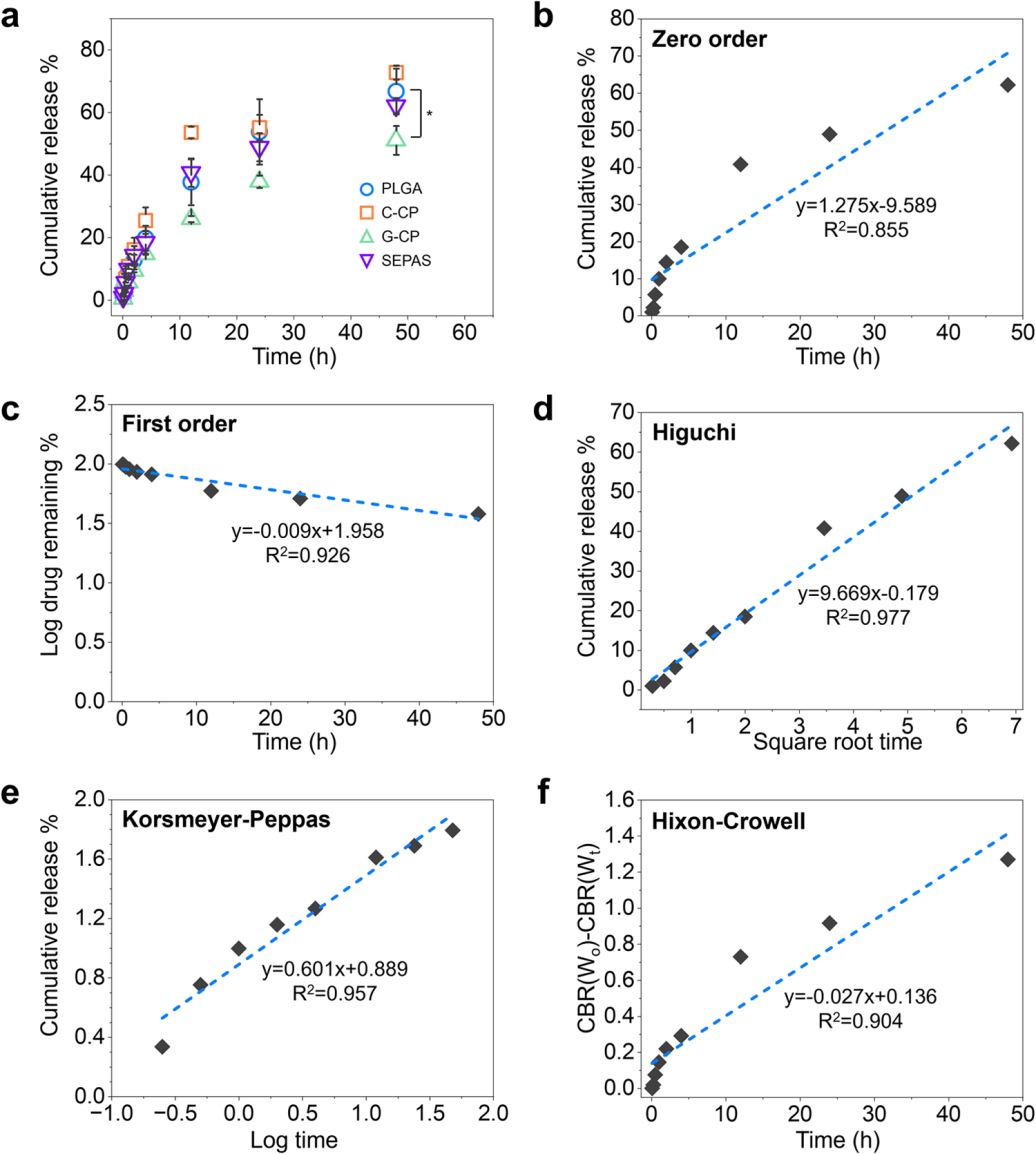
(a) In vitro dexamethasone (DEX) release profile of PLGA, C–CP, G-CP, and SEPAS at pH 6.8 with 0.01 w/v% of α-amylase and 2 w/v% of mucin. Data are expressed as mean ± standard deviations (*n* = 3). Statistical significance between the indicated groups was analyzed using two-tailed Student’s *t* tests. Kinetic model fitting of average Cumulative DEX release from SEPAS NPs using (b) Zero-order, (c) First-order, (d) Higuchi, (e) Korsmeyer–Peppas, and (f) Hixson–Crowell models, where CBR denotes the cube root of the drug amount; CBR­(W_0_) represents the cube root of the initial drug amount, and CBR­(W*_t_*) represents the cube root of the remaining drug amount at time *t*.

In contrast, G-CP showed the slowest release over the entire time window. Considering the strong mucin affinity and aggregation propensity of guanidinium-rich surfaces (Figure 3), this retardation is consistent with adsorption of mucin/salivary biomacromolecules and formation of a diffusion-limiting aggregate layer that reduces the effective release rate of DEX. Because G-CP represents the G-COS-exposed adhesive state generated after starch degradation, this release behavior is also relevant to the postunmasking state of SEPAS near the epithelial interface. After SEPAS adheres in a G-CP state, mucin association on the mucus-facing side may retard outward drug diffusion, while the epithelium-facing side remains close to the target surface, potentially favoring localized DEX release near the epithelial interface. Notably, SEPAS exhibited release kinetics closer to PLGA than to G-CP, suggesting that the starch shield mitigates premature biomacromolecule association and does not act as a persistent barrier to drug release under α-amylase-containing conditions, where partial enzymatic degradation of the shield may further prevent long-lived diffusion resistance.

The release profile was further interpreted in relation to the time scale of mucus penetration and epithelial association. Previous buccal nanoparticle delivery studies have evaluated mucus penetration, epithelial cell uptake, and buccal tissue distribution within a few-hour window, including 2–4 h in ex vivo and in vivo buccal tissue models.^18,46,47^ Based on this time scale, 4 h was selected as a relevant reference point to estimate DEX retention during the transport-to-adhesion process. At 4 h, SEPAS released approximately 18.47% of the loaded DEX, indicating that approximately 81.53% remained within the nanoparticle system. This result suggests that a substantial fraction of DEX is retained during mucus traversal and remains available after α-amylase-triggered exposure of the G-COS layer and subsequent mucoadhesion near the epithelial interface.

To elucidate the release mechanism of SEPAS, the release data were fitted to common kinetic models (Figure 4b–f). Among the tested models, the Higuchi model exhibited the best linear correlation (*R* ^2^ = 0.977), suggesting that diffusion through the matrix is a major contributor to DEX release.^48,49^ The Korsmeyer–Peppas model also showed high linearity (*R* ^2^ = 0.957) with an release exponent *n* ≈ 0.60, suggesting anomalous transport (diffusion-dominated release with additional contributions such as matrix relaxation and/or minor erosion).^50,51^ In comparison, the Hixson–Crowell model exhibited lower correlation (*R* ^2^ = 0.904), implying that changes in particle geometry or surface erosion are not the primary determinants of release under these conditions.^48^ Overall, the kinetic analysis supports that SEPAS releases DEX predominantly via diffusion-controlled transport, accompanied by secondary non-Fickian contributions. Importantly, the dual-regime interfacial design of SEPAS does not markedly alter the intrinsic sustained-release behavior of the PLGA core under the tested conditions.

### 3.4 Biocompatibility and Anti-Inflammatory Activity

To assess the translational feasibility of SEPAS as a buccal drug delivery platform, we evaluated (i) extract-based cytocompatibility in accordance with ISO 10993 and (ii) the anti-inflammatory bioactivity of DEX released from SEPAS using LPS-stimulated RAW264.7 macrophages (Figure 5). In the WST-8 assay using NIH-3T3 fibroblasts (Figure 5a), G-CP showed a modest reduction in cell viability (∼77.8%) compared to the other samples. In contrast, additional starch shielding increased the viability for SEPAS to ∼94.8%. Although the reduced viability of G-CP may be associated with the presence of polycationic chitosan-derived species in the extract, all samples maintained viabilities above 70%, meeting the ISO 10993 cytocompatibility criterion.

**Figure 5:**
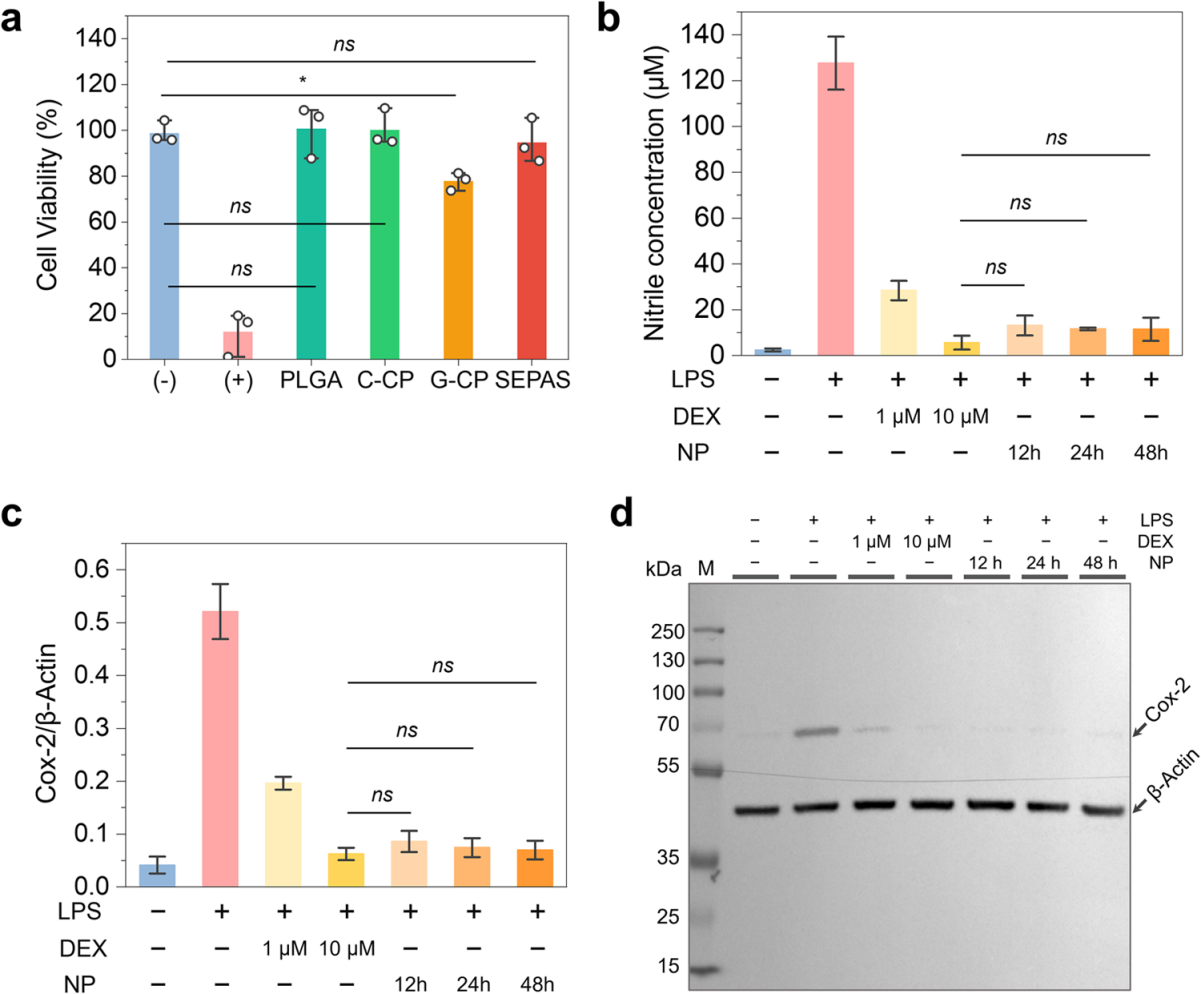
*In vitro* biocompatibility and anti-inflammatory activity of SEPAS NPs. (a) Cytotoxicity toward NIH-3T3 cells assessed by the WST-8 assay following the ISO 10993. Low-density polyethylene and zinc acetate were used as negative and positive controls, respectively. (b) Nitric oxide production (quantified as nitrite concentration), (c) cyclooxygenase-2 (Cox-2) expression (normalized to β-actin) and (d) the corresponding Western blot in RAW264.7 cells in the presence (+) or absence (−) of inflammatory induction with *E. coli*-derived lipopolysaccharide (LPS), anti-inflammatory treatment with 1 and 10 μM DEX, and SEPAS NP dialysates containing DEX released after 12, 24, and 48 h. Data are expressed as means ± standard deviations (*n* = 3). Statistical significance between the indicated groups was analyzed using two-tailed Student’s *t* tests.

We next examined whether DEX released from SEPAS remains pharmacologically active and can suppress inflammatory responses. RAW264.7 macrophages were activated with lipopolysaccharide (LPS), treated with free DEX (1 or 10 μM) as controls, or treated with SEPAS dialysates collected at 12, 24, and 48 h to reflect time-resolved drug release (Figure 5b,c).

NO production was quantified as nitrite (NO_2_^–^) concentration. Under normal conditions, RAW264.7 cells produced ∼2.4 μM nitrite, which markedly increased upon LPS stimulation (∼127.6 μM) and decreased following DEX treatment in a dose-dependent manner (28.4 μM at 1 μM; 5.6 μM at 10 μM). Treatment with SEPAS dialysates also suppressed nitrite production, with later dialysates showing stronger suppression (11.4 μM for the 48 h dialysate), approaching the 10 μM DEX group within experimental variability (Figure 5b; *p* > 0.05 for the indicated comparisons).

At the protein level, Cox-2, a key enzyme in the inflammatory pathway, was measured relative to β-actin as the housekeeping protein. Expression levels were only 0.04-fold under normal conditions, increasing to 0.52-fold after LPS stimulation, and decreasing back to 0.20- and 0.06-fold upon 1 and 10 μM DEX treatment, respectively. SEPAS dialysates also induced down-regulation, decreasing Cox-2 expression from 0.09-fold after 12 h release to 0.07-fold after 48 h release (Figure 5c; *p* > 0.05 for the indicated comparisons). This effect was comparable to that of the 10 μM DEX treatment and significantly different from that of the 1 μM DEX treatment. Overall, these results confirm cytocompatibility of the SEPAS formulation and demonstrate that DEX released from SEPAS retains anti-inflammatory activity over extended release times, consistent with the sustained release profile observed in Figure 4.

## 4 Conclusions

We report salivary enzyme–responsive penetration-to-adhesion switching nanoparticles (SEPAS-NPs) designed to address the penetration–retention trade-off in transmucosal drug delivery. SEPAS-NPs integrate a G-COS adhesive layer with a starch-based, enzyme-degradable shield that masks premature mucin interactions during transport. In mucus-mimicking conditions containing mucin and α-amylase, SEPAS NPs exhibited minimal mucin binding, while enzymatic degradation of the starch layer triggered exposure of the underlying G-COS layer, resulting in enhanced retention at a mucin-coated interface and supporting a sequential transport-then-anchor mechanism. SEPAS-NPs also enabled sustained DEX release with diffusion-dominated kinetics. Extract-based cytocompatibility together with macrophage assays indicated that the formulation is cytocompatible and that released DEX retains anti-inflammatory activity under the tested conditions.

Beyond the buccal model, this enzyme-programmable, dual-regime interfacial strategy provides a broadly applicable framework for transmucosal delivery, where carriers must both traverse mucin-rich barriers and achieve localized retention at the target interface. Because mucus composition, thickness, and turnover vary substantially across mucosal tissues, including oral, nasal, ocular, vaginal, and gastrointestinal mucosa, the SEPAS concept offers a general route to adapt nanocarrier behavior to diverse mucus environments by sequentially regulating transport and anchoring through a degradable protective shell. Accordingly, SEPAS-type switching platforms may enable more reliable delivery in applications where mucus-mediated immobilization and rapid clearance fundamentally limit therapeutic access and efficacy.

## Supplementary Material



## Notes

Synthesis process of *N*-guanidinium chitosan oligosaccharide; nanoparticle fabrication process; potentiometric titration curve, NMR spectra, and FT-IR spectra of *N*-guanidinium chitosan oligosaccharide; ζ-potential of nanoparticles; areal deposit weight of coated nanoparticles; SEM images, photographs, and water contact angle of nanoparticle-coated film; composition of artificial saliva (PDF)
