# Supplementary material for: Salivary Enzyme-Responsive Switching Nanoparticles Overcoming Diffusion and Absorption Barriers for Transmucosal Delivery
Source: ACS Appl Mater Interfaces. 2026 Jun 3;18(24):33618–29. doi: 10.1021/acsami.6c05144 (PMC13308602; doi:10.1021/acsami.6c05144)
Supplement: Supplementary file 1 [file am6c05144_si_001.pdf]

Supporting Information

Salivary Enzyme-Responsive Switching Nanoparticles Overcoming Diffusion and Absorption Barriers for Transmucosal Delivery

Se Kye Park[a,b,†], *Lam Tan Hao*[a,1,†] *Hee Jung Park*[c], *Dong Yun Lee*[b,*], *Hyeonyeol Jeon*[a,d,*], *Hyo Jeong Kim*[a,d,*]

[a] Research Center for Bio-Based Chemistry, Korea Research Institute of Chemical Technology (KRICT), Ulsan, 44429, Republic of Korea

[b] Department of Polymer Science and Engineering, Kyungpook National University, Daegu, 41566, Republic of Korea

[c] Technical Support Center for Chemical Industry, Korea Research Institute of Chemical Technology (KRICT), Ulsan 44429, Republic of Korea

[d] Advanced Materials & Chemical Engineering, Korea National University of Science and Technology (UST), Daejeon 34113, Republic of Korea

† These authors contributed equally to this work.

* Correspondence should be addressed to: dongyunlee@knu.ac.kr; hyjeon@krict.re.kr; khjkye@krict.re.kr

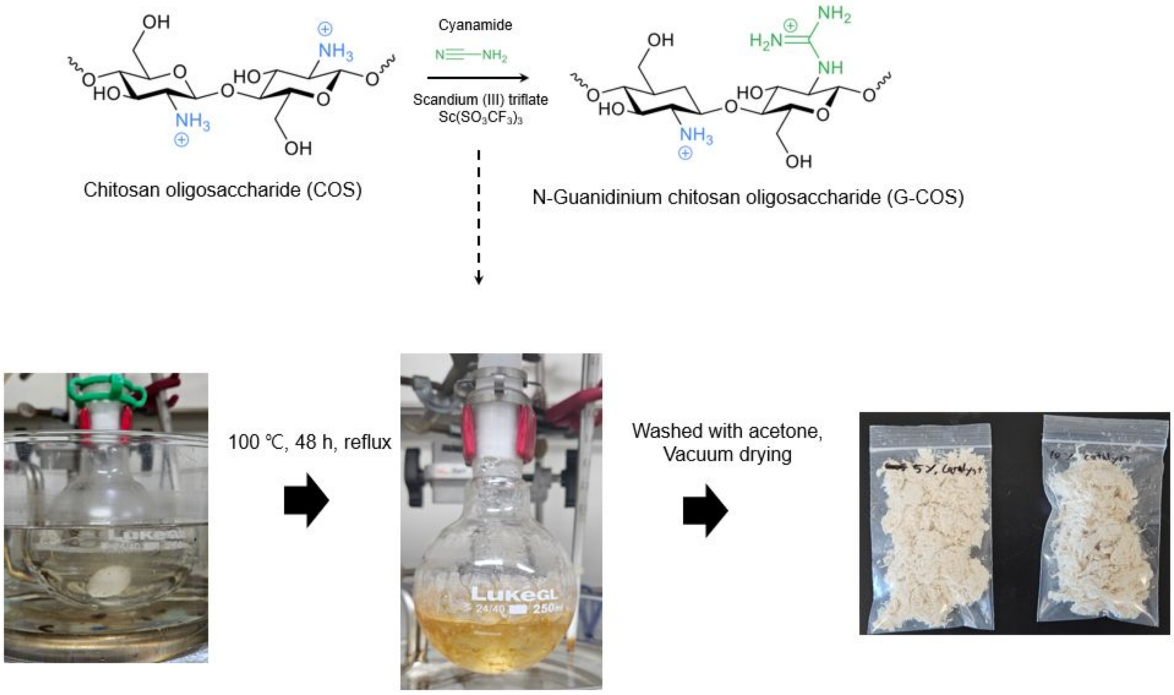

Figure S1. Schematic illustration of the synthesis process of *N*-guanidinium chitosan oligosaccharide.

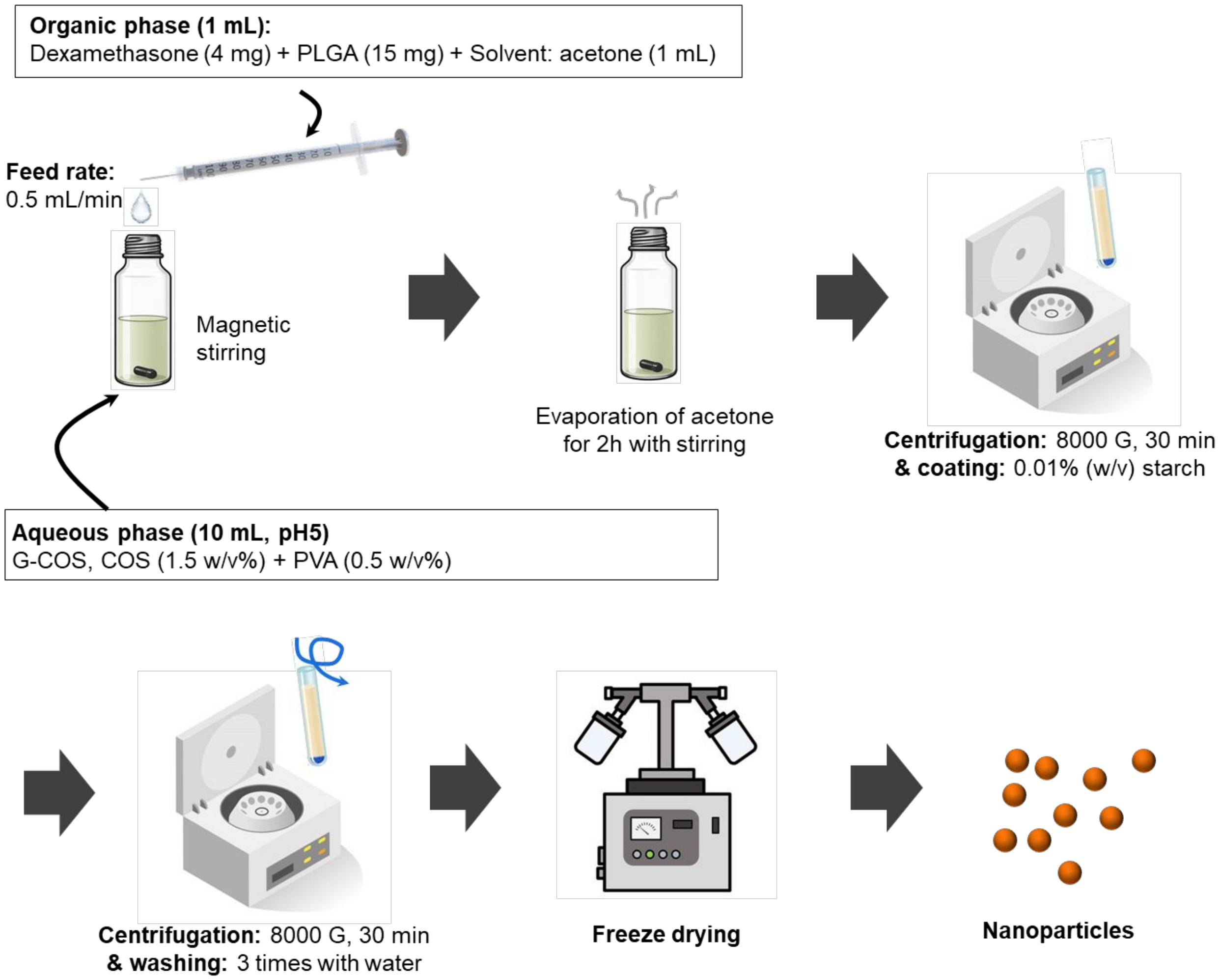

Figure S2. Schematic illustration of the nanoparticle fabrication process.

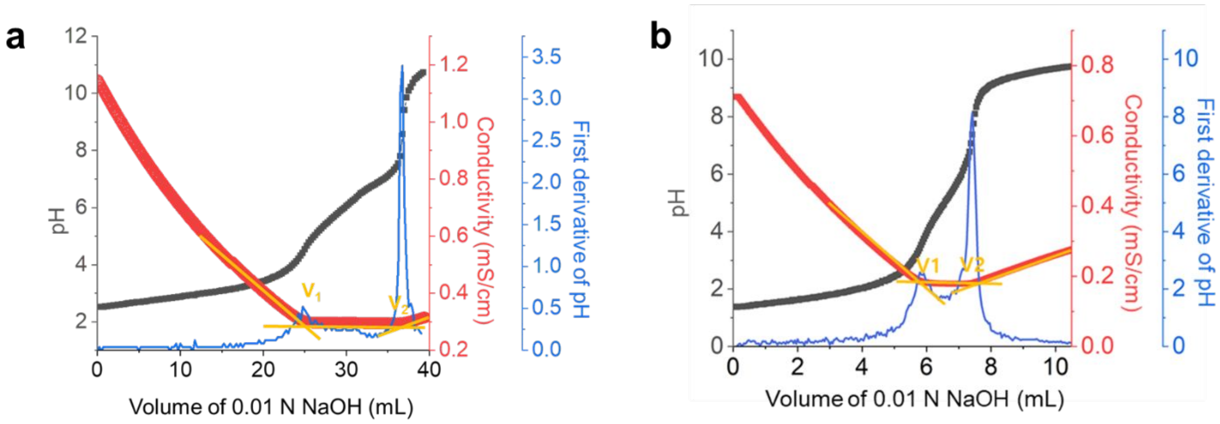

Figure S3. Potentiometric titration curve of (a) chitosan and (b) *N*-guanidinium chitosan (G-COS).

The guanidinium content [Gd^+] of G-COS was quantified using an indirect titration-based approach. Direct determination of [Gd^+] by potentiometric titration was not pursued due to the absence of a distinct equivalence point, which arises from the weak and distributed acid–base behavior of guanidinium groups under dilute titration conditions [1,2]. Consequently, Gd^+ content was evaluated based on changes in the ammonium (NH_3^+) concentration. Specifically, the NH_3^+ contents of the G-COS were independently determined, and the decrease in NH_3^+ after functionalization was attributed to its conversion into guanidinium groups. The difference between these values was used to estimate the amount of Gd^+ incorporated into the COS (Figure S3).

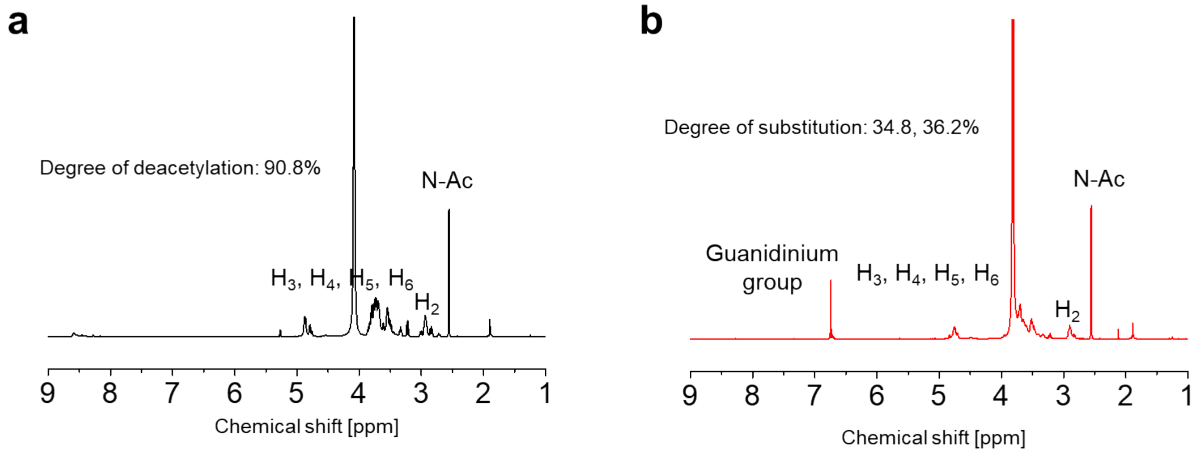

Figure S4. NMR spectra of (a) COS and (b) G-COS, showing characteristic peaks corresponding to guanidinium modification compared with pristine chitosan.

In the COS spectrum (Figure S4a), characteristic chitosan backbone signals were observed, with H3–H6 resonances appearing at 3.5–4.2 ppm and the H2 proton at approximately 3.0 ppm. A weak N-acetyl methyl signal near 2.5 ppm was detected, corresponding to a degree of deacetylation of 90.8%. In the G-COS spectrum (Figure S4b), an additional signal assigned to guanidinium protons appeared between 6.5–7 ppm, confirming successful guanidinium functionalization [3]. The backbone proton signals were retained, indicating structural preservation. Integration analysis yielded a degree of substitution of 34.8–36.2%.

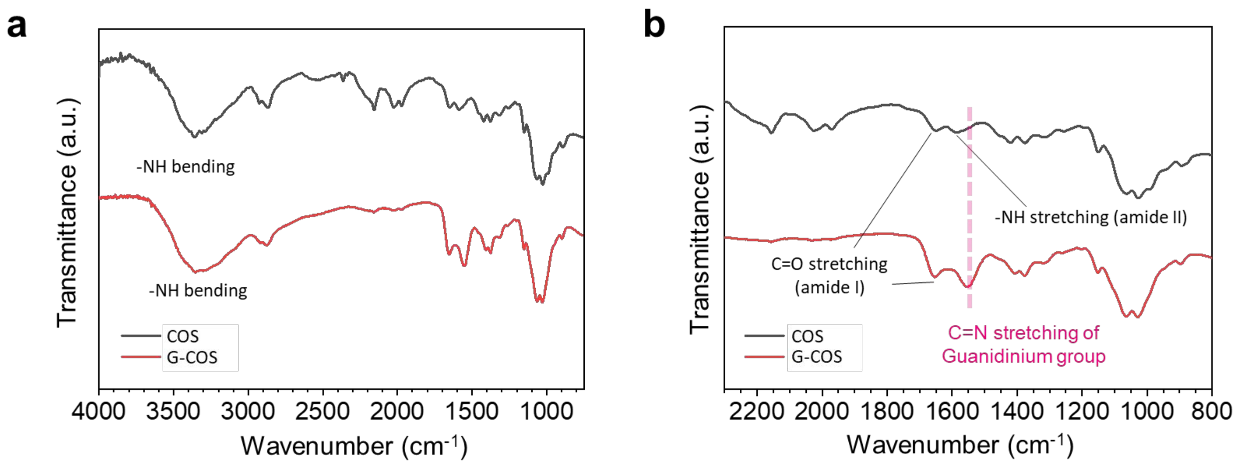

Figure S5. FT-IR spectra of COS and G-COS with (a) full-range and (b) enlarged spectra in the 2200–800 cm^{-1} region.

The FT-IR spectrum of COS exhibits characteristic absorption bands of chitosan, including a broad band at 3200 to 3500 cm^{-1} attributed to N–H and O–H stretching vibrations, as well as bands at 1652 cm^{-1} and at 1593 cm^{-1} corresponding to amide I (C=O stretching of residual N-acetyl groups) and amide II (N–H bending), respectively (Figure S5a). In contrast, the G-COS spectrum shows clear changes in the amide region. In addition to the amide I band, an absorption band at 1552 cm^{-1} is observed, which is assigned to C=N stretching vibrations of the guanidinium group, indicating successful guanidination of COS. (Figure S5b) [4–6].

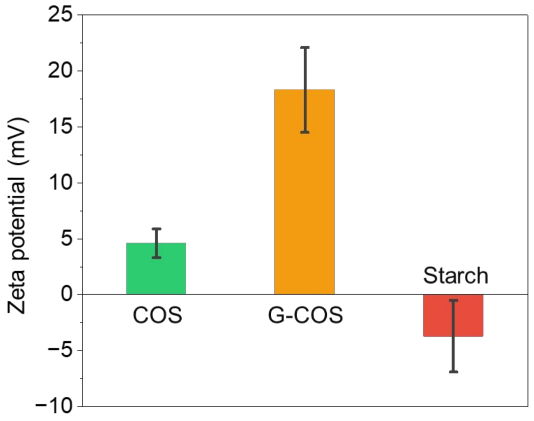

Figure S6. Zeta potential of COS, G-COS and starch measured in PBS at pH 7.4.

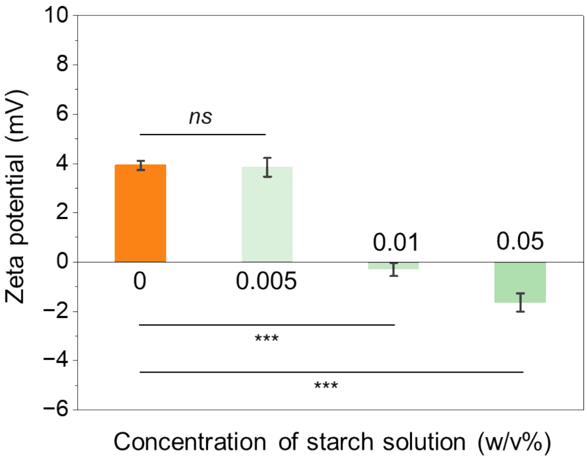

Figure S7. Zeta potential of nanoparticles as a function of starch coating concentration. Data are expressed as means ± standard deviations (n=3). Statistical significance between the indicated groups was analyzed using two-tailed Student's t-tests.

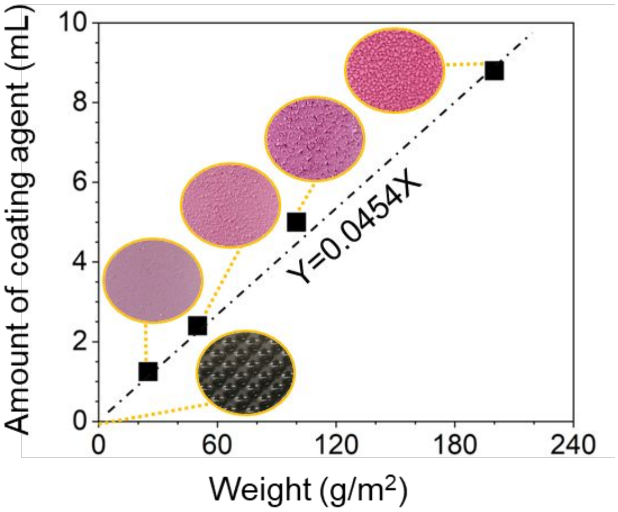

Figure S8. Areal deposit weight of the SEPAS NPs with different amounts of the sprayed coating agent. Insets are surface photographs of samples at the marked amount of the coating agent.

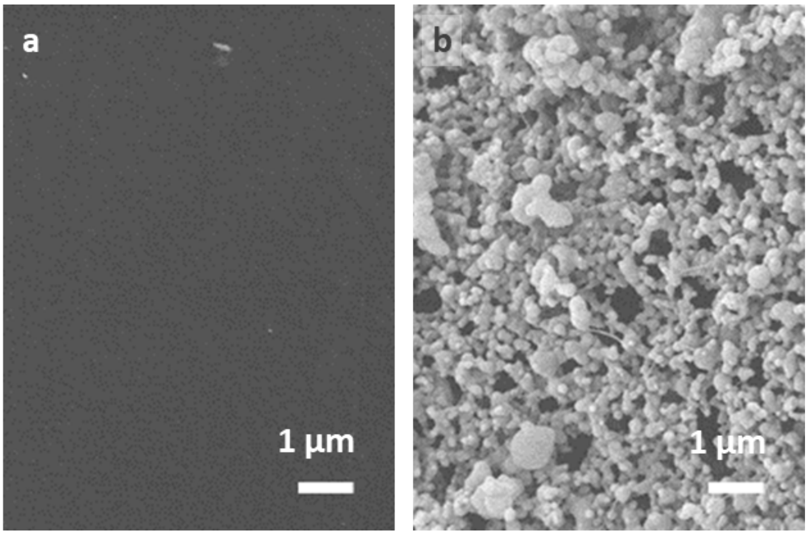

Figure S9. SEM images of bare gelatin films and nanoparticle-coated film. a) bare film and b) SEPAS NP coated film

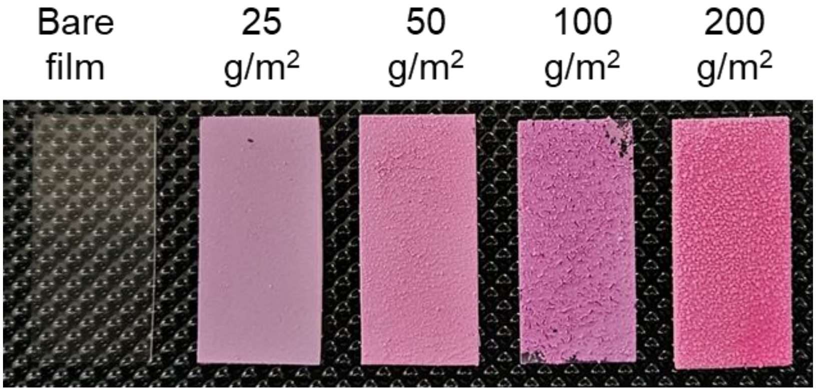

Figure S10. Photographs of gelatin films after SEPAS NP coating with increasing coating amounts (25, 50, 100, and 200 g/m²).

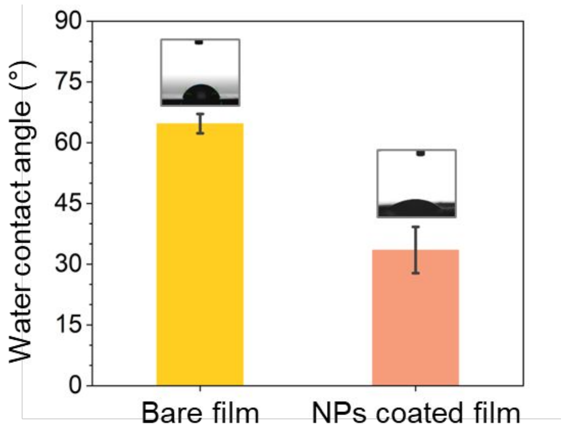

Figure S11. Water contact angle measurements of bare and SEPAS NPs-coated gelatin films.

Table S1. Composition of artificial saliva according to the European Commission Joint Research Centre (JRC)

Component	Chemical Formula	Concentration (mg/L)	Supplier
Magnesium chloride	$MgCl_2$	166.3	Sigma Aldrich (USA)
Calcium chloride	$CaCl_2$	147.0	Sigma Aldrich (USA)
Dipotassium hydrogen phosphate	K_2HPO_4	753.1	Sigma Aldrich (USA)
Potassium carbonate	K_2CO_3	525.1	Sigma Aldrich (USA)
Sodium chloride	NaCl	327.2	Sigma Aldrich (USA)
Potassium chloride	KCl	745.8	Sigma Aldrich (USA)

References

[1] J.C. Masini, O.E.S. Godinho, L.M. Aleixo, Determination of ionizable groups of proteins by potentiometric titration in concentrated solutions of guanidine hydrochloride, Fresenius J. Anal. Chem. 360 (1998) 104–111. https://doi.org/10.1007/s002160050651.

[2] C. Giroud, M. Moreau, T.A. Mattioli, V. Balland, J.-L. Boucher, Y. Xu-Li, D.J. Stuehr, J. Santolini, Role of Arginine Guanidinium Moiety in Nitric-oxide Synthase Mechanism of Oxygen Activation, Journal of Biological Chemistry 285 (2010) 7233–7245. https://doi.org/https://doi.org/10.1074/jbc.M109.038240.

[3] A. Salama, M. Hasanin, P. Hesemann, Synthesis and antimicrobial properties of new chitosan derivatives containing guanidinium groups, Carbohydr. Polym. 241 (2020) 116363. https://doi.org/10.1016/j.carbpol.2020.116363.

[4] P. Sahariah, B.M. Óskarsson, M.Á. Hjálmarsdóttir, M. Másson, Synthesis of guanidinylated chitosan with the aid of multiple protecting groups and investigation of antibacterial activity, Carbohydr. Polym. 127 (2015) 407–417. https://doi.org/https://doi.org/10.1016/j.carbpol.2015.03.061.

[5] N.F. Khan, H. Nakamura, H. Izawa, T. Ikeda, S. Ifuku, M. Otagiri, M. Anraku, Guanidinylated Chitosan as a Multifunctional Enhancer for Improved Flurbiprofen Delivery, Biol. Pharm. Bull. 48 (2025) 1246–1254. https://doi.org/10.1248/bpb.b25-00394.

[6] B. Divband, M. Aghazadeh, Z.H. Al-qaim, M. Samiei, F.H. Hussein, A. Shaabani, S. Shahi, R. Sedghi, Bioactive chitosan biguanidine-based injectable hydrogels as a novel BMP-2 and VEGF carrier for osteogenesis of dental pulp stem cells, Carbohydr. Polym. 273 (2021) 118589. https://doi.org/https://doi.org/10.1016/j.carbpol.2021.118589.
